# The THO/TREX Complex Active in Alternative Splicing Mediates Plant Responses to Salicylic Acid and Jasmonic Acid

**DOI:** 10.3390/ijms222212197

**Published:** 2021-11-11

**Authors:** Nengxu Sun, Xiangjiu Kong, Yueyan Liu, Tingting Gong, Xiaoyong Gu, Lijing Liu

**Affiliations:** The Key Laboratory of Plant Development and Environmental Adaptation Biology, Ministry of Education, School of Life Sciences, Shandong University, Qingdao 266237, China; 201932352@mail.sdu.edu.cn (N.S.); xjkong0528@sdu.edu.cn (X.K.); liuyy9702@163.com (Y.L.); aligtt@163.com (T.G.); guxy18@sdu.edu.cn (X.G.)

**Keywords:** salicylic acid, jasmonic acid, THO/TREX complex, alternative splicing, induced defense

## Abstract

Salicylic acid (SA) and jasmonic acid (JA) are essential plant immune hormones, which could induce plant resistance to multiple pathogens. However, whether common components are employed by both SA and JA to induce defense is largely unknown. In this study, we found that the *enhanced disease susceptibility 8* (*EDS8*) mutant was compromised in plant defenses to hemibiotrophic pathogen *Pseudomonas syringae pv. maculicola* ES4326 and necrotrophic pathogen *Botrytis cinerea*, and was deficient in plant responses to both SA and JA. The *EDS8* was identified to be *THO1*, which encodes a subunit of the THO/TREX complex, by using mapping-by-sequencing. To check whether the EDS8 itself or the THO/TREX complex mediates SA and JA signaling, the mutant of another subunit of the THO/TREX complex, THO3, was tested. *THO3* mutation reduced both SA and JA induced defenses, indicating that the THO/TREX complex is critical for plant responses to these two hormones. We further proved that the THO/TREX interacting protein SERRATE, a factor regulating alternative splicing (AS), was involved in plant responses to SA and JA. Thus, the AS events in the *eds*8 mutant after SA or JA treatment were determined, and we found that the SA and JA induced different alternative splicing events were majorly modulated by EDS8. In summary, our study proves that the THO/TREX complex active in AS is involved in both SA and JA induced plant defenses.

## 1. Introduction

Plants, as sessile organisms, have developed a multilayer immune system to cope with environmental pathogens. After pathogen infection, plants have the ability to recognize pathogen-associated molecular patterns (PAMPs) to induce PAMP-triggered immunity (PTI) and/or sense effectors secreted by pathogens to induce effector-triggered immunity (ETI) at the infection site. In the distant parts that are not infected, plants also induce systemic acquired resistance (SAR) to render the whole plant more resistant to the following infection [1]. Phytohormones play critical roles in the plant immune process, and salicylic acid (SA) and jasmonic acid (JA) have been identified as essential plant immune-related hormones to regulate PTI, ETI, and SAR [2,3]. In general, SA mediates defenses against biotrophic and hemibiotrophic pathogens that get nutrients from living plant tissues, while JA has been shown to restrict the growth of necrotrophic pathogens which live on dead cells for nutrients [4,5].

Both SA and JA signaling pathways have been well explored. SA is sensed by the no expressor of pathogen-related (NPR) family proteins including NPR1, NPR3, and NPR4, which interact with TGA transcription factors to regulate the expression of *pathogen-related* (*PR*) genes, such as *PR1* [6,7,8]. JA isoleucine (JA-Ile), the active form of JA, is accumulated during pathogen infection and is perceived by its receptor coronatine insensitive 1 (COI1), which interacts with and promotes the degradation of jasmonate zim domain (JAZ) proteins to release the MYC transcription factors to trigger the expression of JA responsive genes, such as *plant defensin 1.2 (PDF1.2)* [9,10]. As both SA and JA regulate plant immunity, their crosstalk has been widely studied. In recent years, multiple genes whose mutation differently alter plant responses to SA and JA have been reported. For example, overexpression of WRKY70 results in constitutive expression of SA responsive genes, while JA responsive genes are upregulated in the *WRKY70* RNA interference line [11]; The JA signaling is repressed, but SA signaling is activated in the mutant of *resistant to phytophthora 5* [12]. The discoveries of these genes support the antagonistic crosstalk between SA and JA, which is consistent with SA and JA mediate plant resistance to opposite pathogens. In turn, the mechanism of SA/JA antagonism will be better understood by the further functional exploration of these genes.

Even the antagonistic crosstalk between SA and JA is well documented, some studies also support a synergistic relationship of these two hormones, especially in induced resistance [3,12,13,14,15]. For example, it has been reported that the Oligogalacturonides could induce plant resistance to *Pseudomonas syringae pv. tomato* DC3000 (*Pst* DC3000) by triggering both SA and JA signaling [14]; Foliar treatment of JA, then SA, triggers the strongest resistance against tobacco mosaic virus in *Nicotiana benthamiana* compared with only SA or JA application [3]. Additionally, even SA and JA have been connected with pathogens with different lifestyles, both SA and JA treatments could activate a common defense system and both induce plant resistance to *Pst* DC3000 [16,17]. However, whether common components or different players are recruited by SA and JA to induce defense is largely unknown.

To answer this question, we searched the published reports to check if mutants that may compromise in both SA and JA signaling could be identified, and the *enhanced disease susceptibility 8* (*EDS8*) mutant aroused our interest. The *eds8* mutant was firstly isolated in a forward genetic screen for ethyl methanesulfonate (EMS) mutants that exhibit increased susceptibility to hemibiotrophic pathogen *Pseudomonas syringae pv. maculicola* (*Psm*) ES4326 [18]. Later, Kim et al. found the enhanced susceptibility of *eds8* to be correlated with reduced expression of *PR1,* indicating that EDS8 may play a role in SA signaling in defense [19]. The *eds8* mutant also shows less induction of JA responsive gene *PDF1.2* and compromised protection to *Pst* DC3000 by JA treatment, which suggests EDS8 is required for plant response to JA [20,21]. However, whether EDS8 regulates both SA and JA induced defenses needs to be further confirmed, and the gene encoding EDS8 needs to be identified.

In this study, we proved EDS8 is required for plant defenses to both biotrophic and necrotrophic pathogens and for plant responses to SA and JA. Mapping-by-sequencing determined that a mutation in *suppressor of the transcriptional defects of Hpr1 mutants by overexpression 1 (THO1)*, which encodes a component of THO/TREX complex, was introduced into the *eds8* mutant. The THO/TREX complex is conserved in yeast, plants, and animals [22]. In plants, it has been shown to regulate plant responses to stresses including phosphate starvation and aluminum toxin [22]. The involvement of THO/TREX complex in SA and JA signaling was further confirmed by checking the mutant phenotypes of another THO/TREX component, THO3. The TREX/THO complex regulates multiple cellular processes including alternative splicing (AS) which may rely on the interaction of THO/TREX complex with serrate (SE). The *se* mutant also reduced plant responses to SA and JA, and the different alternative splicing (DAS) events in WT induced by SA and JA were compromised in the *eds8* mutant. This study identified a key player in both SA and JA responses and may pave the way to explore the mechanism of induced resistance shared by SA and JA.

## 2. Results

### 2.1. EDS8 Mutation Compromised Plant Response to SA

**Figure 1:**
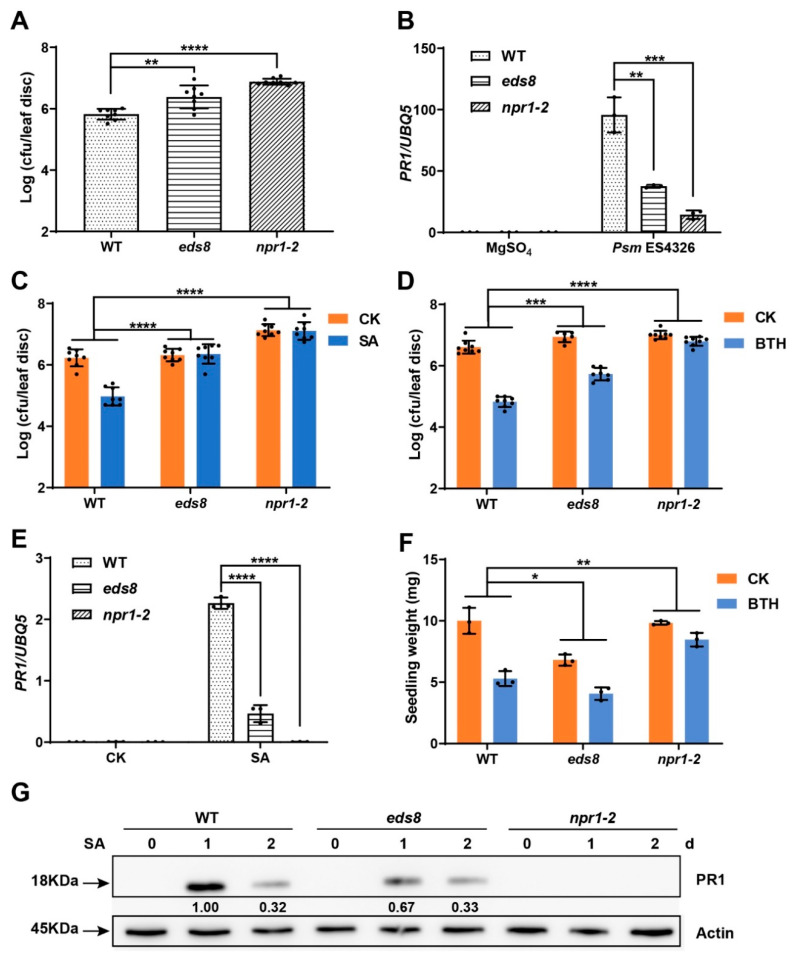
SA related phenotypes of the *eds8* mutant. (**A**) The *eds8* mutant was more susceptible to *Psm* ES4326. Three-week-old plants were inoculated with *Psm* ES4326 (OD_600_ = 0.0001) and pathogen growth was determined three days post inoculation (dpi). Significant difference was detected using Student’s *t*-test. Data are shown as mean ± SD (*n* = 8). (**B**) The expression of *PR1* after *Psm* ES4326 inoculation. Samples were collected 1 dpi. Significant difference was detected using Student’s *t*-test. Data are shown as mean ± SD (*n* = 3). SA (**C**) or BTH (**D**) induced resistance to *Psm* ES4326 in WT, *eds8,* and *npr1* mutants. Three-week-old plants were sprayed with SA, BTH or water one day before pathogen infiltration (OD_600_ = 0.001), and pathogen growth was determined three days later. Significant difference was detected by two-way ANOVA. Data are shown as mean ± SD (*n* = 8). (**E**) SA induced expression of *PR1* in WT, *eds8,* and *npr1* mutants. Twelve-day-old seedlings were sprayed with SA or water, and samples were collected one day after treatment. Significant difference was detected using Student’s *t*-test. Data are shown as mean ± SD (*n* = 3). (**F**) BTH induced growth inhibition assay. Ten-day-old seedlings were treated with 600 μM BTH or water (CK) for five days, and then the seedlings were weighed. Significant difference was detected by two-way ANOVA. Data are shown as mean ± SD (*n* = 3). (**G**) The protein levels of PR1 in WT, *eds8,* and *npr1* mutants after SA treatment. Twelve-day-old seedlings were sprayed with SA or H_2_O (CK) and samples were collected at 0, 1, or 2 days after treatment for Western blot using anti-PR1 antibody. The relative PR1 levels in different samples were compared with PR1 level in WT sample treated with SA for one day. The levels of Actin were also detected as internal controls. All these experiments were repeated three times with similar results. * *p* < 0.05; ** *p* < 0.01; *** *p* < 0.001; **** *p* < 0.0001.

To investigate if the *eds8* mutant changed the plant response to both SA and JA, we ordered the *eds8* seeds from SALK institute and firstly checked its increased susceptibility to hemibiotrophic pathogen *Psm* ES4326. The third and fourth leaves of three-week-old plants were inoculated with *Psm* ES4326, and three days later, the infected leaves were used for measuring pathogen growth. Under our growth conditions, the *eds8* mutant was, even not as susceptible as the mutant of SA receptor NPR1, more susceptible than wild type (WT) (Figure 1A). The induction of *PR1* by *Psm* ES4326 was also compromised in the *eds8* mutant, which is correlated with its disease susceptibility to *Psm* ES4326 (Figure 1B).

Based on these results, we wonder if EDS8 modulates SA accumulation. Meanwhile, as shown in Figure S1, an equal amount of free SA and even more SAG were detected in *eds8* compared with in WT, which cannot explain the lower expression of *PR1* in *eds8*. Then, we further determined if the plant response to SA was altered in the *eds8* mutant. The propagation of *Psm* ES4326 cells in leaves of the *eds8* mutant that were sprayed with SA one day before pathogen infection was analyzed. Notably, SA was unable to induce defense to *Psm* ES4326 in the *eds8* mutant as efficiently as in WT (Figure 1C). SA-induced *PR1* expression was also markedly suppressed in the *eds8* mutant compared with in WT (Figure 1E). Correspondingly, PR1 protein was also less accumulated in *eds8* mutant than in WT after SA treatment (Figure 1G and Figure S2). Benzothiadiazole (BTH) is a chemical analogue of SA, which induces plant defense dependent on SA signaling and causes a substantial reduction in plant biomass after a long-term treatment [23]. Consistently, the BTH induced resistance was also partially dependent on EDS8 (Figure 1D). We further examined the BTH-induced reduction in plant biomass. As shown in Figure 1F, the BTH treatment reduced the weight of the seedling from 10 to 5.3 mg in WT. However, the growth inhibition of the seedling caused by BTH was compromised in the *eds8* mutant (from 6.8 to 4.1 mg). All these results support the notion that EDS8 is required for plant response to SA.

### 2.2. EDS8 Mutation Compromised Plant Response to JA

**Figure 2:**
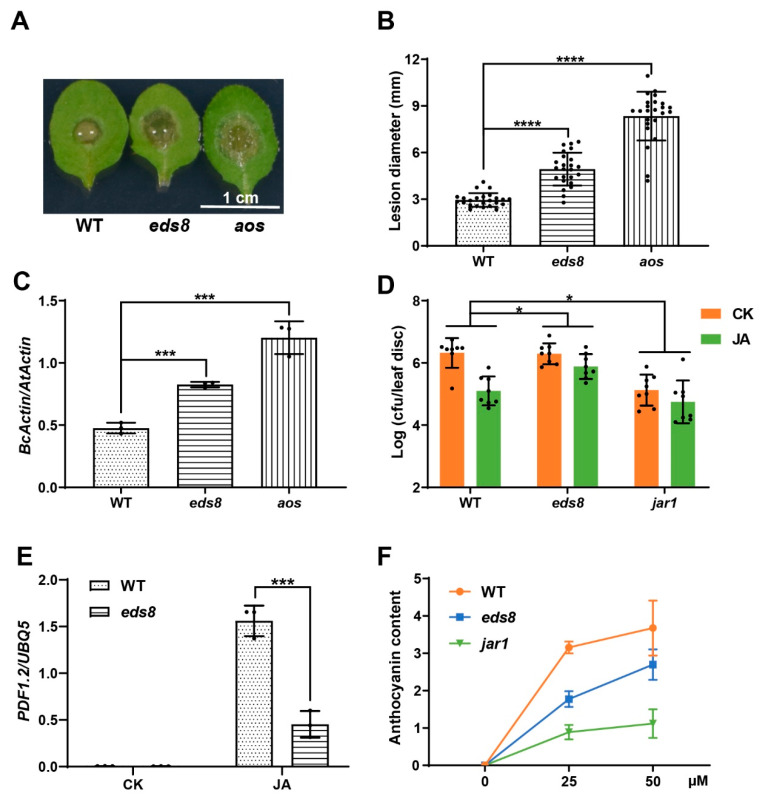
JA related phenotypes of the *eds8* mutant. (**A**–**C**) The *eds8* mutant was more susceptible to *Botrytis cinerea*. Third and fourth true leaves of three-week-old plants were inoculated with the spores of *Botrytis cinerea* (10^5^ spores/mL). Photos were taken (**A**), lesion sizes were measured with ImageJ (**B**) and DNA was extracted for qPCR (**C**) at 36 h after pathogen inoculation. Significant difference was detected using Student’s *t*-test. Data are shown as mean ± SD (*n* = 24). (**D**) JA induced resistance to *Psm* ES4326 in WT, *eds8,* and *jar1* mutants. Three-week-old plants were sprayed with JA or H_2_O (CK) one day before pathogen infiltration (OD_600_ = 0.001), and pathogen growth was determined three days later. Significant difference was detected by two-way ANOVA. Data are shown as mean ± SD (*n* = 8). (**E**) JA induced expression of *PDF1.2* in WT and *eds8* mutants. Twelve-day-old seedlings were sprayed with JA or H_2_O (CK), and samples were collected one day after treatment. Significant difference was detected using Student’s *t*-test. Data are shown as mean ± SD (*n* = 3). (**F**) JA induced anthocyanin accumulation assay. Seeds were sowed onto 1/2 MS plates with a different concentration of JA, and the anthocyanin accumulation was determined 14 days later. Data are shown as mean ± SD (*n* = 3). All these experiments were repeated three times with similar results. * *p* < 0.05; *** *p* < 0.001; **** *p* < 0.0001.

To further confirm the role of EDS8 in plant response to JA, we checked the response of the *eds8* mutant to a necrotrophic pathogen, *Botrytis cinerea,* as JA signaling has been shown to be essential for plant defense to it [24,25]. The third and fourth true leaves of three-week-old WT and *eds8* plants were inoculated with *Botrytis cinerea* spores, and the mutant of JA synthesis gene, *AOS*, was applied as a control. Two days later, larger lesion size was observed on drop-inoculated *eds8* leaves than on WT leaves (Figure 2A,B). Consistently, more *Botrytis cinerea* DNA was detected in the *eds8* mutant than in WT (Figure 2C).

As the levels of JA and JA-Ile, the active form of JA, were comparable in *eds8* and in WT (Figure S3), the response of *eds8* to JA was further determined in our experimental conditions. We checked the protective effect of JA in the *eds8* mutant to *Psm* ES4326 infection with *JA resistant 1* (*JAR1*) mutant serving as the control [26]. Three-week-old WT, *eds8,* and *jar1* mutants were foliar sprayed with JA one day prior to inoculation with *Psm* ES4326 (Figure 2D). JA treatment strongly reduced pathogen growth by 13.5-fold in WT, but not in *jar1* as expected, while no protection was observed in the *eds8* mutant (Figure 2D). JA induced expression of *PDF1.2* was also compromised in *eds8* compared with in WT plants (Figure 2E). Long-term JA treatment causes anthocyanin accumulation in leaves [27]. Thus, seeds were sowed onto 1/2 MS plates with different concentration of JA, and the accumulation of purple pigmentation, which indicates the accumulation of anthocyanin, was measured 14 days later. As shown in Figure 2F, the anthocyanin contents were much lower in the *eds8* mutant than in WT under JA treatment. Taken together, the defense assays, gene expression analyses, and anthocyanin accumulation measurements consistently demonstrate that EDS8 plays a critical role in plant response to JA.

### 2.3. Identification of EDS8 Gene Using Mapping-by-Sequencing

**Figure 3:**
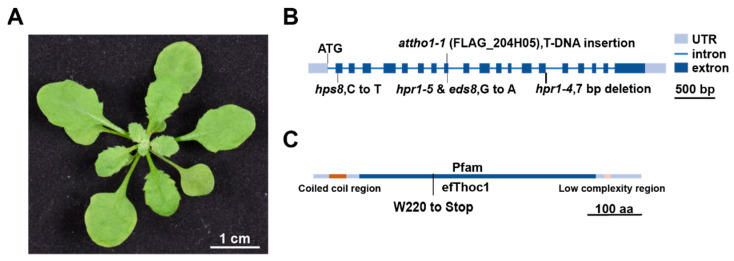
The *eds8* mutant is positional cloned to *THO1* using mapping-by-sequencing. (**A**) The serrated leaf phenotype of the *eds8* mutant. (**B**) Schematic representation of the *EDS8* gene. Untranslated regions (UTR) are represented as grey boxes, exons as dark blue boxes and introns as blue lines. The positions and mutation forms of *eds8*, *hpr1-5* and other previous published mutants are indicated. (**C**) Schematic representation of EDS8 protein. The mutation site of *eds8* is marked. The coiled coil regain of EDS8 protein is represented by red box, the Pfam efThoc1 domain predicted by SMART by dark blue box, and low complexity region by pink box.

The *eds8* mutant has been isolated for more than 20 years, while the underlying mutation has not been identified. To clone the candidate gene that is responsible for *eds8* phenotype, we backcrossed *eds8* to Columbia WT and separated the F2 populations into two groups, the *eds8*-like group and the WT-like group, based on the serrated leaves phenotype of *eds8* (Figure 3A). Then, DNA samples extracted from Columbia WT, the *eds8* mutant, the pool of *eds8*-like F2 plants, and the pool of WT-like F2 plants were used for mapping-by-sequencing [28]. We determined that a mutation at chromosome 5, position 3,068,296, was responsible for the serrated leaves phenotype of *eds8.* A G-to-A mutation occurred at this locus, which results in a nonsynonymous transition that replaced a codon for tryptophan (TGG) with a stop codon (TGA) in exon 8 of At5g09860 (Figure 3B). Thus, instead of full length EDS8 protein (also named THO1 and HPR1), a truncated version of it was produced in the *eds8* mutant (Figure 3C).

**Figure 4:**
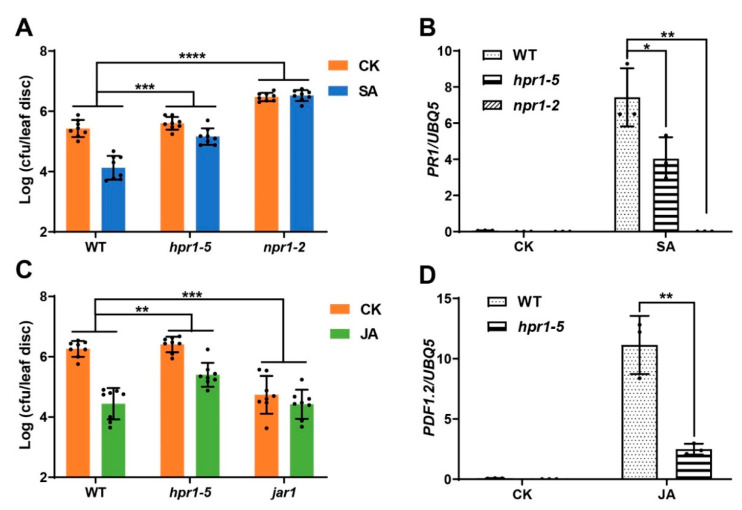
The phenotypes of SA and JA induced defenses in another *eds8* mutant allele, *hpr1-5*. (**A**) SA induced resistance to *Psm* ES4326 in WT, *hpr1-5,* and *npr1* mutants. Significant difference was detected by two-way ANOVA. Data are shown as mean ± SD (*n* = 8). (**B**) SA induced expression of *PR1* in WT, *hpr1-5,* and *npr1* mutants. Significant difference was detected using Student’s *t*-test. Data are shown as mean ± SD (*n* = 3). (**C**) JA induced resistance to *Psm* ES4326 in WT, *hpr1-5* and *jar1* mutants. Significant difference was detected by two-way ANOVA. Data are shown as mean ± SD (*n* = 8). (**D**) JA induced expression of *PDF1.2* in WT and *eds8* mutants. Significant difference was detected using Student’s *t*-test. Data are shown as mean ± SD (*n* = 3). All these experiments were conducted as previously described and repeated three times with similar results. * *p* < 0.05; ** *p* < 0.01; *** *p* < 0.001; **** *p* < 0.0001.

The *At5g09860* encodes a component of the THO/TREX complex and has been identified in several studies to regulate plant responses to stresses including phosphate starvation, aluminum resistance, and plant basal defense [22,29,30,31,32]. To further confirm that *At5g09860* is the underlying gene for the SA and JA insensitive phenotypes of the *eds8* mutant, we used another mutant allele of *At5g09860*, *hpr1-5,* which has the same mutation as *eds8,* to check if it showed similar responses to SA and JA [33]. Three-week-old plants were pretreated with SA or JA, and then *Psm* ES4326 was inoculated as previously described. Consistent with in the *eds8* mutant, the pathogen growths were also less restricted in *hpr1-5* after SA or JA treatment than in WT (Figure 4A,C). The inductions of SA and JA responsive genes were also compromised in the *hpr1-5* mutant (Figure 4B,D). Our results demonstrate that *At5g09860* is the underlying gene of *eds8*.

### 2.4. THO3, Another Subunit of THO/TREX Complex, Positively Regulated Plant Response to SA and JA

**Figure 5:**
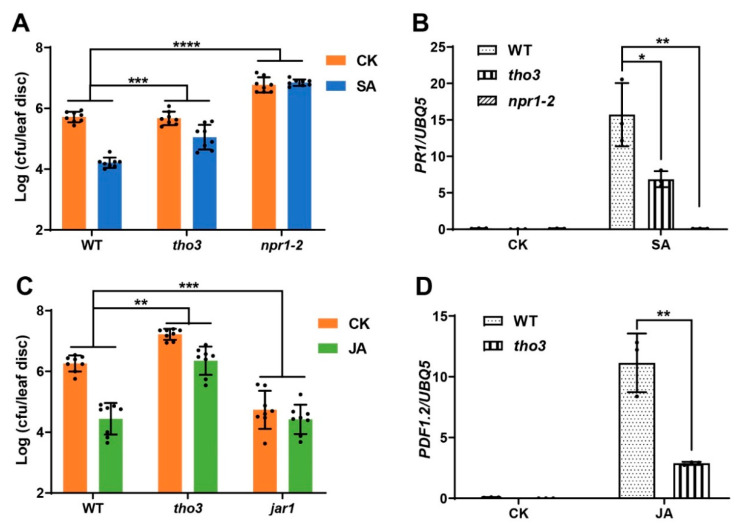
The phenotypes of SA and JA induced defenses in the mutant of *THO3*, encoding another subunit of THO/TREX complex. (**A**) SA induced resistance to *Psm* ES4326 in WT, *tho3,* and *npr1* mutants. Significant difference was detected by two-way ANOVA. Data are shown as mean ± SD (*n* = 8). (**B**) SA induced expression of *PR1* in WT, *tho3,* and *npr1* mutants. Significant difference was detected using Student’s *t*-test. Data are shown as mean ± SD (*n* = 3). (**C**) JA induced resistance to *Psm* ES4326 in WT, *tho3,* and *jar1* mutants. Significant difference was detected by two-way ANOVA. Data are shown as mean ± SD (*n* = 8). (**D**) JA induced expression of *PDF1.2* in WT and *tho3* mutants. Significant difference was detected using Student’s *t*-test. Data are shown as mean ± SD (*n* = 3). All these experiments were conducted as previous described and repeated three times with similar results. * *p* < 0.05; ** *p* < 0.01; *** *p* < 0.001; **** *p* < 0.0001.

As EDS8 is a component of the THO/TREX complex, we wonder whether EDS8 regulates the plant response to SA and JA represents a function of this complex. To solve this problem, a mutant of another THO/TREX component needs to be tested. In Arabidopsis, four components of THO/TREX complex (THO1, THO2, THO3 and THO6) are encoded by a single gene [34]. Among these four genes, the *tho2* mutant is lethal, the seedling of the *tho3* mutant has increased root hairs, similar with the *eds8* mutant, while the *tho6* mutant shows a WT-like amount of root hair [32]. Thus, the *tho3* mutant was applied in this study. As shown in Figure 5A,B, the SA induced *PR1* expression and plant resistance to *Psm* ES4326 were compromised in the *tho3* mutant. Similar results were obtained for JA induced response in *tho3* as in the *eds8* mutant, indicating that THO3 is also required for plant response to JA (Figure 5C,D). These results suggest that the THO/TREX complex positively regulates plant responses to SA and JA.

### 2.5. The THO/TREX Interacting Protein SERRATE Modulated Plant Responses to SA and JA

**Figure 6:**
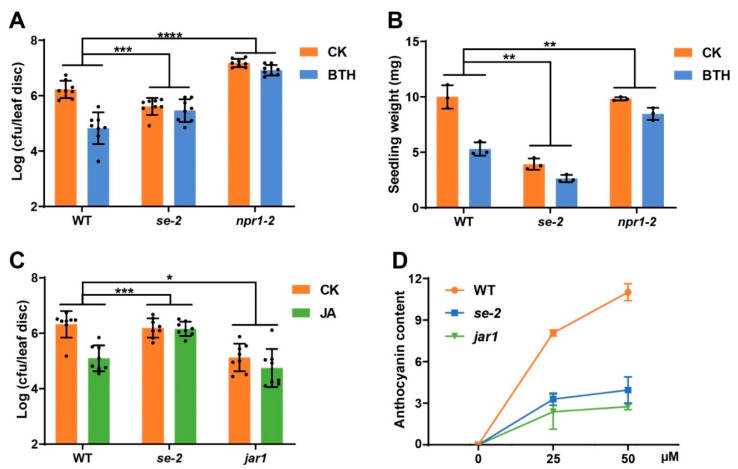
The phenotypes of SA and JA induced responses in the *se* mutant. (**A**) BTH induced resistance to *Psm* ES4326 in WT, *se,* and *npr1* mutants. Data are shown as mean ± SD (*n* = 8). (**B**) BTH induced growth inhibition assay in WT, *se,* and *npr1* mutants. Data are shown as mean ± SD (*n* = 3). (**C**) JA induced resistance to *Psm* ES4326 in WT, *se,* and *jar1* mutants. Data are shown as mean ± SD (*n* = 8). (**D**) JA induced anthocyanin accumulation assay in WT, *se,* and *jar1* mutants. Data are shown as mean ± SD (*n* = 3). All these experiments were conducted as previously described and repeated three times with similar results. Significant difference was detected by two-way ANOVA. * *p* < 0.05; ** *p* < 0.01; *** *p* < 0.001; **** *p* < 0.0001.

Serrate (SE) is a conserved RNA effector that has well known function in alternative splicing (AS), and its mutation causes a serrated leaf phenotype as the *eds8* mutant [35]. A recent study showed that SE interacted with multiple components of the THO/TREX complex, which has also been shown to affect plant mRNA processing, including AS [36,37,38]. Thus, THO/TREX may regulate plant response to SA/JA by modulating AS. To test this possibility, we firstly checked the SA and JA responses in the *se* mutant. Compared with WT, the *se* mutant showed insensitive phenotypes to SA and JA (Figure 6). The SA or JA induced defense to *Psm* ES4326 is significantly lower in the *se* mutant than in WT (Figure 6A,B). Furthermore, the BTH-triggered reduction in plant biomass and JA-triggered anthocyanin accumulation were also compromised in the *se* mutant (Figure 6C,D). The THO/TREX complex and SE also function in small RNA processing, while in our hand, the mutation of *hyponastic leaves 1* (*HYL1*), which physically interacts with SE to fine-tune the small RNA processing [39,40], did not significantly affect plant responses to SA and JA (Figures S4 and S5). These results clearly suggest that THO/TREX complex mediates the plant responses to SA and JA through modulating AS.

### 2.6. The SA and JA Induced Different Alternative Splicing Were Dependent on EDS8

**Figure 7:**
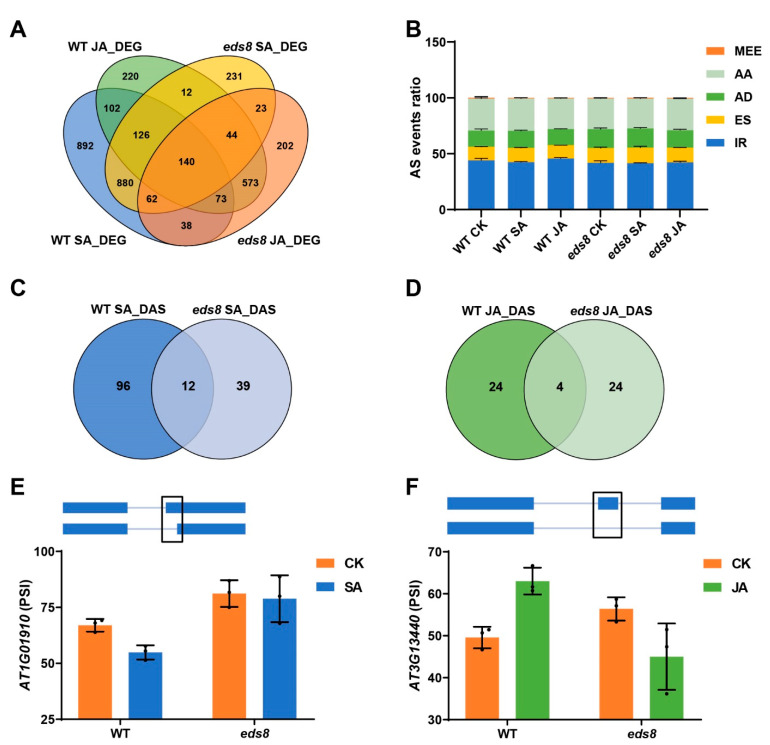
EDS8 influences SA and JA induced changes in gene expression and alternative splicing (AS). Twelve-day-old seedlings were treated with/without SA or JA, and samples were collected for full length RNA sequencing. (**A**) Venn diagrams of different expressing genes induced by SA and JA in WT and the *eds8* mutant. (**B**) The different AS types for AS events in all samples. MEE, Mutually exclusive exons; AA, Alternative acceptor sites; AD: Alternative donor sites; ES, Exon skipping; IR, Intron retention. (**C**) Venn diagrams of different alternative splicing (DAS) genes in WT and *eds8* mutant after SA treatment. (**D**) Venn diagrams of DAS genes in WT and *eds8* mutant after JA treatment. (**E**) Representative EDS8 dependent and SA regulated DAS event (Event region NC003070.9:313419-313566 of *At1g01910*). (**F**) Representative EDS8 dependent and JA regulated DAS event (Event region NC003074.8:4378075-4378587 of *At3g13440*). Exons are represented as blue boxes, and introns as blue lines. PSI, percent spliced in.

To further prove that the THO/TREX complex modulates SA and JA signaling through its active in AS, we detected the AS events in WT and the *eds8* mutant by full-length mRNA sequencing. Twelve-day-old seedlings of WT and the *eds8* mutant treated with/without SA or JA for 4 h were used to examine the SA and JA regulated different alternative splicing (DAS), and the dependence of DAS on EDS8. Three biological replicates for each genotype and treatment were generated. We firstly analyzed the transcriptome changes after SA or JA treatment. As shown in Figure 7A, the expression of 2313 genes and 1290 genes were modulated by SA and JA, respectively, and the regulations of 1105 genes by SA and 460 genes by JA were dependent on EDS8. These RNA-Seq data further supported the important role of EDS8 in SA and JA signaling.

Then, the AS events of different samples were analyzed. Generally, 1137 to 1734 AS events were detected in all samples, and these events could be classified into several types such as intron retention (IR), exon skipping (ES), alternative donor sites (AD), and alternative acceptor sites (AA) (Figure 7B and Table S1). The ratios of different AS types were comparable in WT and the *eds8* mutant and in samples with/without hormone treatment (Figure 7B). A total of 108 DAS genes (determined by |ΔPSI| ≥ 10%; FDR < 0.05), represented 125 AS events, were detected after SA treatment (Figure 7C and Figure S6). EDS8 was indispensable for the regulation of SA on these DASs (Figure 7C and Figure S7A). For example, AA was induced by SA for the eighth exon of *At1g01910* in WT but not in the *eds8* mutant (Figure 7E). These SA regulated and EDS8-dependent DASs were involved in the molecular functions of binding, catalytic activity, and transporter activity (Figure S8A). We further analyzed the DAS genes after JA treatment and identified 24 EDS8-dependent JA regulated DAS genes, such as *At3g13440*, *At1g02840,* and *At3g17920* (Figure 7D,F and Figure S7B). The JA induced ES of the second exon of *At3g13440* was observed in WT but not in the *eds8* mutant (Figure 7F). These genes were also majorly overrepresented in the molecular functions in binding, catalytic activity, and transporter activity (Figure S8). Our data clearly proved that the THO/TREX complex is required for SA- and JA-regulated AS.

## 3. Discussion

Previous studies suggest that both SA and JA could induce plant resistance to multiple pathogens [16,41,42,43,44], while little is known about the common factors used by these two hormones. In this study, we found that EDS8 mediated the plant response to both SA and JA. EDS8 was positionally cloned to THO1, a component of the THO/TREX complex, and THO3, another subunit of the THO/TREX complex, also participated in the plant response to SA and JA and proved that the THO/TREX complex plays a role in plant induced resistance by these hormones. The THO/TREX complex has been reported to regulate plant responses to stresses including phosphate starvation, aluminum toxin, and is also involved in plant ethylene signaling and plant basal defense [22,29,30,31,32,33]. Thus, we expanded the functions of THO/TREX complex in this study.

The THO/TREX complex is conserved in yeast, animals, and plants. In plants, it has been reported to modulate pre-mRNA processing, such as AS and mRNA export, and the accumulation of small RNAs [29,30,32,38,45,46,47]. As the mutation of *THO3* does not impair mRNA export [38], we checked the SA and JA responses in the mutant of *SE*, which is a THO/TREX interacting protein and has well documented roles in AS and small RNA accumulation [35,36]. From our data, we predict the THO/TREX complex active in AS mediates SA and JA signaling in plants. We cannot exclude the roles of small RNA accumulation and mRNA export in SA and JA induced defenses mediated by other proteins/complexes, as these processes have been shown to be involved in plant defense [48,49]. Combined our results with the previous findings that the THO/TREX complex modulates plant response to aluminum toxin and phosphate starvation through its active in mRNA export, and/or small RNA biosynthesis [29,32], we proposed that the activities of the THO/TREX complex in different cellular processes are critical to plant responses to different stresses.

In Arabidopsis, multiple genes undergo alternative splicing, which increases the protein diversity by selection of splicing sites, intron retention, or exon skipping of the pre-mRNAs to regulate plant developments and stress responses [50,51,52,53]. AS plays crucial functions in plant growth, development, and stress responses including plant immunity. The important role of AS in plant defense has been reported in multiple species such as Sorghum, maize, wheat, sugarcane, tobacco, and Arabidopsis [54,55,56,57,58,59,60]. For example, plant elicitor peptides (Peps)-induces PTI by regulating AS of calcium-dependent protein kinase 28 (CPK28) though disturbing the interaction of immunoregulatory RNA-binding protein (IRR) with the CPK28 pre-mRNA in Arabidopsis [61]; Suppressor of *abi3-5* (SUA) and required for *snc4-1D* 2 (RSN2) control the splicing of receptor-like kinase (RLK), like the suppressor of *npr1-1*, constitutive 4 (SNC4) and chitin elicitor receptor kinase 1 (CERK1), to regulate the plant defense mediated by RLKs [62]. Our results add another layer of AS regulation to plant defense by changing the immune-related hormone signaling.

The important role of AS in plant response to JA has been well characterized. JAZ family proteins are the key transcription repressors of JA signaling whose JAS domain is required for the degradation of JAZs by COI1 to initiate plant response to JA [9]. AS results in *JAZ* variants translated to protein without JAS domain and constitutively repress JA signaling [63]. The AS of JAZ is mediated by pre-mRNA-processing protein 39a (PRP39a) and PRP40a [64], while multiple genes other than JAZs also undergo AS after JA treatment in previous report and this study [65]. *EDS8* mutation altered the splicing of a subset of mRNAs after JA treatment, indicating that the THO/TREX complex is also in charge of AS in plant response to JA. Less studies have been performed to explore the role of AS on SA signaling. Apple NPR1-like genes undergo AS after pathogen infection, which suggests that the AS of them may contribute to apple disease resistance [66]. In this study, we checked the SA induced DAS for the first time and found a critical regulator for this process. Meanwhile, how these DAS genes are involved in SA or JA signaling needs to be studied in the future. In summary, we identified that EDS8 encodes a subunit of the THO/TREX complex, which regulates SA and JA signaling through its activity in AS.

## 4. Materials and Methods

### 4.1. Plant Materials and Growth Conditions

*Arabidopsis thaliana* plants used in this study were all in the Columbia-0 (Col-0) background. The *eds8* mutant was purchased from the SALK collection. Seeds of *tho3,* and *hpr1-5* were gifts from Dr. Qiguang Wen. Seeds of *se-2* and *hyl1-2* were kindly provided by Dr. Dong Liu. The seeds were surface sterilized with 75% ethanol for 1 min and washed with sterile water once; then disinfected with 1% sodium hypochlorite for 8–10 min and washed with sterile water for three times. After 4 °C treatment for 2 days, seeds were grown on 1/2 MS agar plates or in pots under 22 °C and a 12-h light/dark photoperiod with 60% relative humidity conditions. 

### 4.2. Pathogenic Bacteria Inoculation and Growth Determination

*Psm* ES4326 was streaked onto King’s B medium agar plate containing 50 μg/mL streptomycin 36 h prior to infection and incubated at 28 °C. Bacterial suspension (OD_600_ = 0.001) in 10 mM MgSO_4_ was infiltrated into the third and fourth true leaves of three-week-old Arabidopsis using a needless syringe. Quantifications of *in planta* bacterial growth were performed at 3 days post inoculation (dpi). A total of 16 surface-sterilized leaf discs (5 mm diameter) excised from 16 leaves of 8 plants per genotype were randomly separated into 8 pools, and then subjected to the quantification of leaf bacteria. For BTH, SA and JA induced resistance assays, plants were sprayed with BTH (120 µM in water), SA (1 mM in water) or MeJA (100 µM in 0.1% ethanol) 24 h before inoculation. Control plants were sprayed with water.

### 4.3. Botrytis Cinerea Bioassays

*Botrytis cinerea* was grown for 10–14 days in darkness at 22 °C on V8 medium (360 mL V8 juice, 0.2% CaCO_3_, 20% agar). Spores were harvested in water and filtered through miracloth to remove hyphae and were suspended at a final concentration of 1 × 10^5^ spores mL^−1^ in PDA liquid medium. Before inoculation, the third and fourth true leaves of three-week-old Arabidopsis were placed in petri dishes with 0.8% agar. A 5 µL droplet of spore suspension was deposited on the adaxial surface of each leaf. Thereafter, the culture dishes were placed into a growth chamber (22 °C and 12-h light/dark photoperiod) for 36 h and the diameters of the lesions caused by *Botrytis cinerea* were measured using ImageJ.

### 4.4. Growth Inhibition Assays Following BTH Treatment

Ten-day-old seedlings grown in soil were sprayed with 600 μM BTH or water. Five days after treatment, seedlings were collected and weighed. Three repetitions, 10 seedlings for each, were measured for every sample.

### 4.5. Anthocyanin Accumulation Assays

Anthocyanin accumulation assays were performed as described previously [67]. Briefly, seeds were sowed onto 1/2 MS plates supplemented with 0 μM, 25 μM, or 50 μM MeJA. Fourteen days later, seedlings were collected and weighed. Then, seedlings were incubated in extraction buffer (methanol containing 1% HCl) for 24 h at 4 °C in the dark. After centrifugation for 5 min at 12,000 g, the supernatant was assayed spectrophotometrically by determining the absorbance at 530 nm and 657 nm. Relative anthocyanin content was quantified by (A_530_ − 0.25 × A_657_) per gram fresh weight.

### 4.6. Gene Expression Analyses by qPCR

For the detection of the expression changes of marker genes after SA or JA treatment, 12-day-old seedlings from 1/2 MS agar plates were sprayed with 1 mM SA, 100 μM MeJA or water and were collected at the indicated time points. Total RNA was extracted using TRIzol Reagent (Takara) and treated with RQ1 DNase (Promega) according to the manufacturer’s instructions. First-strand cDNA was synthesized from 500 ng of purified total RNA by using the PrimeScript RT Master Mix (Takara). qPCR was performed with a QuantStudio 5 Real-Time PCR Systems (Thermo Fisher Scientific, Waltham, MA, USA) using SYBR Green Real-Time PCR Master Mix (Mei5bio) as recommended by the manufacturer. Target genes were quantified with specific primer pairs listed in Table S2. Gene expression levels were normalized to *UBIQUITIN 5* (*UBQ5*, *At4G05320*). Three technical replicates were performed for each sample.

### 4.7. Protein Extraction and Immunoblots Analysis

For the detection of PR1 protein, 12-day-old seedlings grown on MS media were sprayed with 1 mM SA for 1 or 2 days and 25 seedlings were collected for each sample, frozen in liquid nitrogen and then ground into powder. Protein was extracted with lysis buffer (50 mM Tris (pH 7.5), 150 mM NaCl, 0.1% Triton X-100, 0.2% Nonidet P-40 and protease inhibitor cocktail (Roche)). After centrifugation 12,000× *g* at 4 °C for 5 min, proteins were mixed with the protein loading buffer and were boiled for 10 min at 95 °C before being loaded onto SDS-PAGE gel. Immunoblotting analysis was realized using a primary rabbit polyclonal anti-PR1 antibody (Agrisera, diluted 1:5000) and a secondary anti-rabbit IgG-HRP (EASYBIO, diluted 1:10,000). Anti-β-Actin antibody was used as an internal control.

### 4.8. Mapping-by-Sequencing Analyses

To create mapping populations, the *eds8* mutant was backcrossed with Col-0. The F2 progenies, from the selfing of a single F1 plant, were divided into two groups, the *eds8*-like group and the WT-like group, based on the serrated leaves phenotype of *eds8*. Leaf tissues from F2 individuals in each group were combined in approximately equal amounts (5 mm disc) to obtain approximately equimolar DNA populations. DNA was extracted from samples and the libraries were prepared, and whole genome sequencing was performed through the Illumina platform. By comparing the SNPs of the two F2 groups with *eds8* mutant and Col-0, the mutation site of *eds8* was determined.

### 4.9. Transcriptome Sequencing and Alternative Splicing Analysis

Twelve-day-old WT and *eds8* seedlings were soaked in 1/2 MS liquid with or without SA/JA for 4 h. Three biological replicates (0.3–0.5 g) for each treatment/genotype were collected. The samples were frozen in liquid nitrogen, and then sent to Biomarker Technologies for RNA extraction, cDNA library construction, and RNA sequencing with PromethION platform. Raw reads were filtered with minimum average read quality score = 7 and minimum read length = 500 bp. Full-length, non-chimeric transcripts were determined by mapping to TAIR 10.1 with minimap 2, and AS events were identified using the AStalavista tool.

## Data Availability

All data generated by this study is available upon request.

## Supplementary Materials

The following are available online at https://www.mdpi.com/article/10.3390/ijms222212197/s1.
