# Supplementary material for: The THO/TREX Complex Active in Alternative Splicing Mediates Plant Responses to Salicylic Acid and Jasmonic Acid
Source: Int J Mol Sci. 2021 Nov 11;22(22):12197. doi: 10.3390/ijms222212197 (PMC8619553; doi:10.3390/ijms222212197)
Supplement: Supplementary file 1 [file ijms-22-12197-s001.zip › ijms-1454145-supplementary.pdf]

International Journal of
Molecular Sciences

MDPI

SUPPLEMENTAL MATERIALS

The THO/TREX Complex Active in Alternative Splicing Mediates Plant Responses to Salicylic Acid and Jasmonic Acid

Nengxu Sun †, Xiangjiu Kong †, Yueyan Liu, Tingting Gong, Xiaoyong Gu and Lijing Liu *

The Key Laboratory of Plant Development and Environmental Adaptation Biology, Ministry of Education, School of Life Sciences, Shandong University, 266237 Qingdao, China; 201932352@mail.sdu.edu.cn (N.S.); xjkong0528@sdu.edu.cn (X.K.); liuyy9702@163.com (Y.L.); aligtt@163.com (T.G.); guxy18@sdu.edu.cn (X.G.)

* Correspondence: ljliu@sdu.edu.cn

† These authors contributed equally to the article.

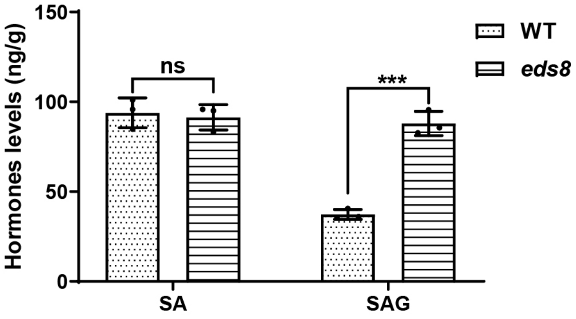

Figure S1. The levels of SA and SAG in WT and *eds8* mutant. The third and fourth true leaves of three-week-old plants were collected. The levels of SA and SAG were measured. Significant difference was detected by student *t*-test. Data are shown as mean ± SD (n = 3). ***, $p < 0.001$; ns, no significant difference.

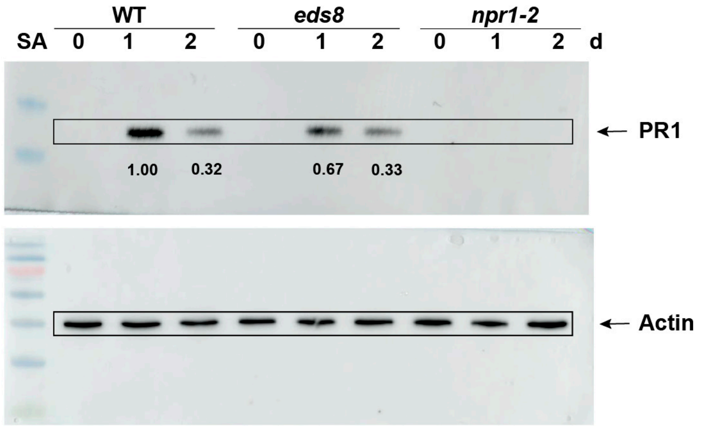

Figure S2. The full scan data for protein levels of PR1 in WT, *eds8* and *npr1* mutants after SA treatment.

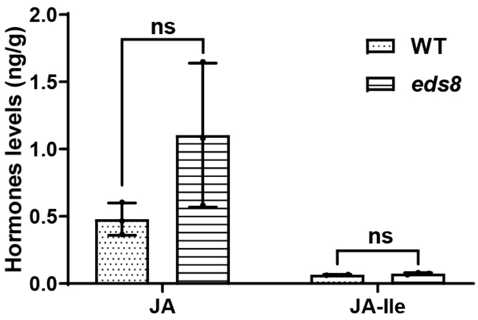

Figure S3. The levels of JA and JA-Ile in WT and *eds8* mutant. The third and fourth true leaves of three-week-old plants were collected. The levels of JA-Ile and JA were measured. Significant difference was detected by student *t*-test. Data are shown as mean ± SD (n = 3). ns, no significant difference.

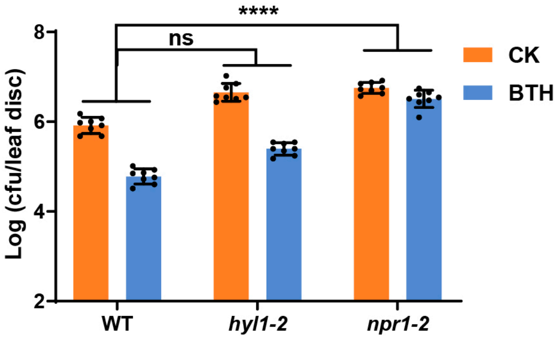

Figure S4. The BTH induced defense in WT, *hyl* and *npr1* mutants. Three-week-old plants were sprayed with BTH or CK (H_2O) one day before pathogen infiltration (OD_{600} = 0.001), and pathogen growth was determined three days later. Significant difference was detected by two-way ANOVA. Data are shown as mean ± SD (n = 8). The experiment was repeated three times with similar results. ****, $p < 0.0001$; ns, no significant difference.

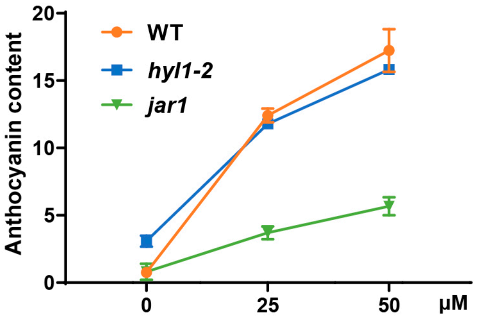

Figure S5. The JA induced anthocyanin accumulation assay in WT, *hyl* and *jar1* mutants. Seeds were sowed onto 1/2 MS plates with different concentration of JA, and the anthocyanin accumulation was determined 14 days later. The experiment was repeated three times with similar results.

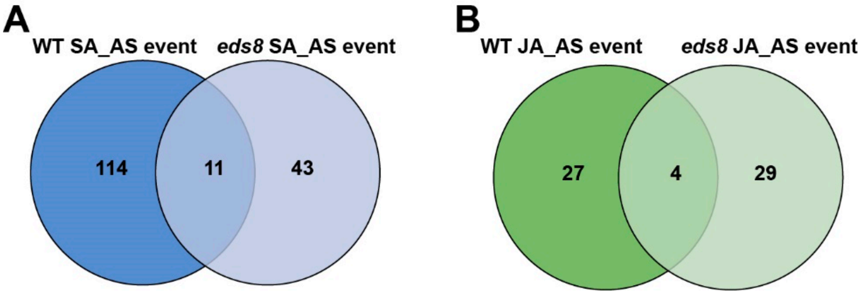

Figure S6. EDS8 influences the SA and JA induced changes on alternative splicing (AS) events. The Venn diagrams of different AS events induced by SA (**A**) and JA (**B**) in WT and *eds8* mutant.

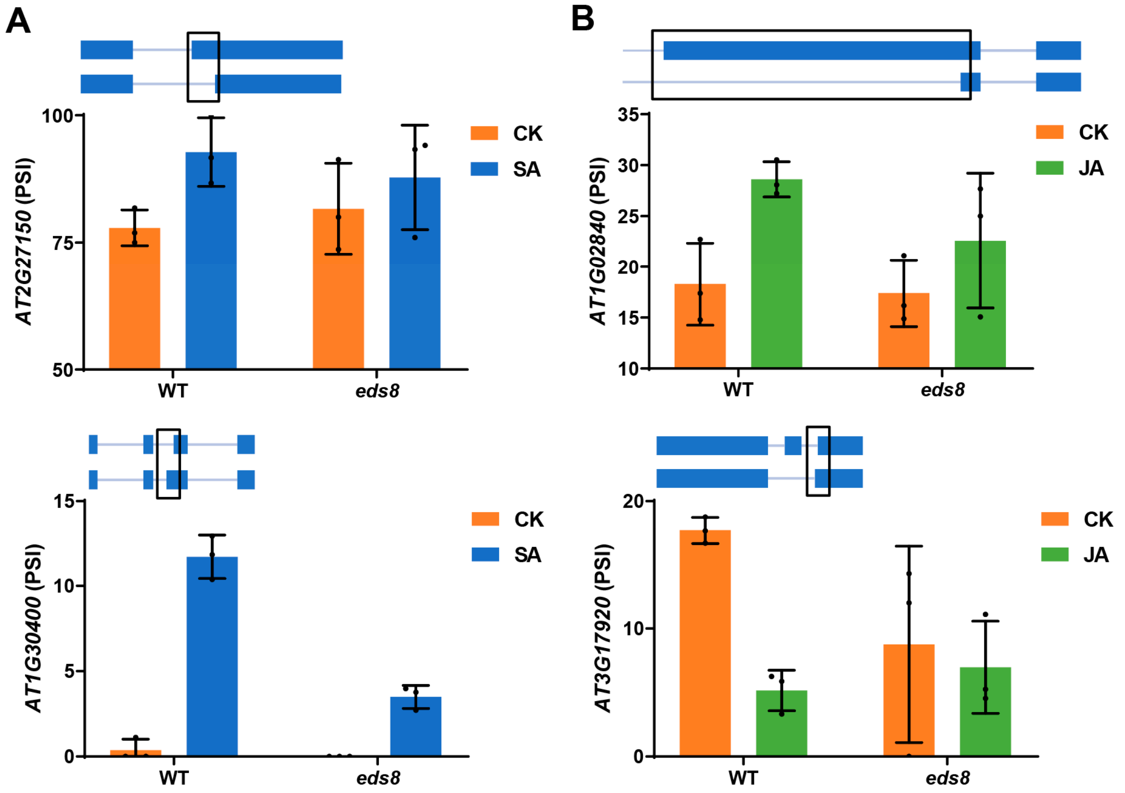

Figure S7. Representative EDS8 dependent DAS events regulated by SA and JA. (**A**) Representative EDS8 dependent DAS events (Event Region NC003071.7:11602079-11602429 of *At2g27150* and NC003070.9:10731068-10731226 of *At1g30400*) regulated by SA. (**B**) Representative EDS8 dependent DAS events (Event Region NC003070.9:628470-629287 of *At1g02840* and NC003074.8:6137696-6137982 of *At3g17920*) regulated by JA. Exons are represented as blue boxes, and introns as blue line. PSI, percent spliced in.

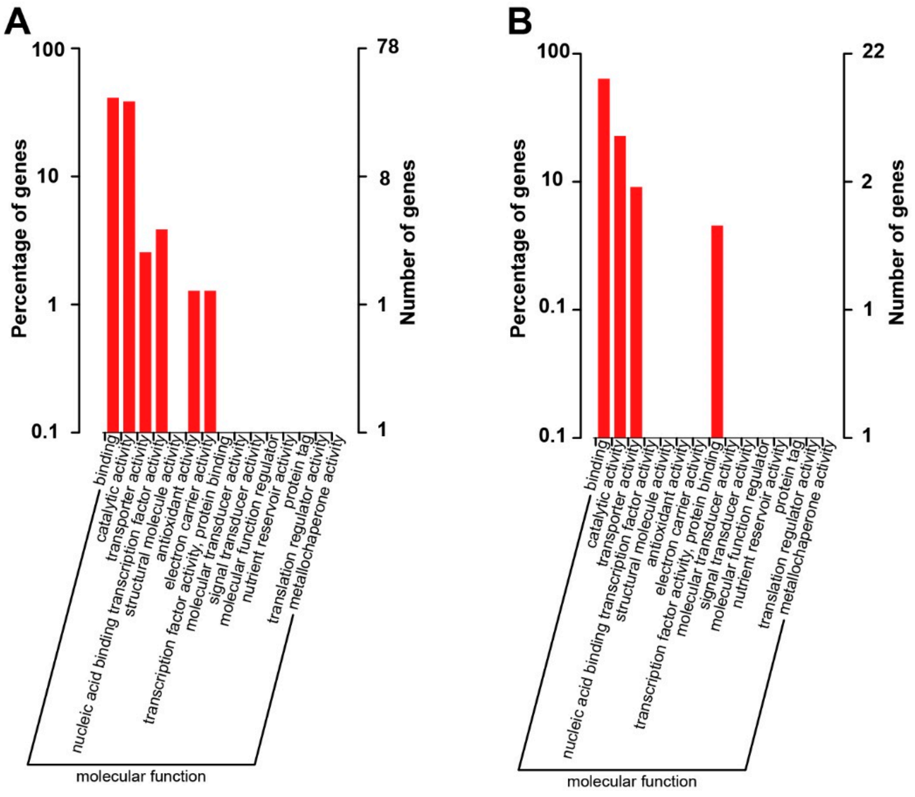

Figure S8. Go terms of the EDS8 dependent SA and JA induced DAS genes. (**A**) GO terms of EDS8 dependent SA induced DAS genes. (**B**) GO terms of EDS8 dependent JA induced DAS genes.

Table S1. The AS events in different samples used for full length mRNA sequencing.

AS type / Sample	IR	ES	AD	AA	MEE
WT CK	516	138	171	333	8
WT SA	560	169	202	382	8
WT JA	544	140	171	324	7
eds8 CK	555	172	224	364	8
eds8 SA	652	219	270	417	11
eds8 JA	538	166	199	359	8

Table S2. The primers used in this study.

Primer	Sequence (5'-3')
UBQ5qPCRFw	GTAAACGTAGGTGAGTCC
UBQ5qPCRRev	GACGCTTCATCTCGTCC
PR1qPCRFw	CTCATACACTCTGGTGGG
PR1qPCRRev	TTGGCACATCCGAGTC
PDF1.2qPCRFw	TTGCTGCTTTCGACGCA
PDF1.2qPCRRev	TGTCCCACTTGGCTTCTCG
AtActin2-1Fw	GGCGATGAAGCTCAATCCAAACG
AtActin2-1Rev	GGTCACGACCAGCAAGATCAAGACG
BcActin-1Fw	TCCAAGCGTGGTATTCTTACCC
BcActin-1Rev	TGGTGCTACACGAAGTTCGTTG
